# Environmental Factors Affecting the Community of Methane-oxidizing Bacteria

**DOI:** 10.1264/jsme2.ME21074

**Published:** 2022-03-26

**Authors:** Hiromi Kambara, Takahiro Shinno, Norihisa Matsuura, Shuji Matsushita, Yoshiteru Aoi, Tomonori Kindaichi, Noriatsu Ozaki, Akiyoshi Ohashi

**Affiliations:** 1 Department of Civil and Environmental Engineering, Graduate School of Engineering, Hiroshima University, 1–4–1, Kagamiyama, Higashi-Hiroshima, Hiroshima 739–8527, Japan; 2 Faculty of Geosciences and Civil Engineering, Kanazawa University, Kakuma-machi, Kanazawa, 920–1192, Japan; 3 Agricultural Technology Research Center, Hiroshima Prefectural Technology Research Institute, 6869, Hara, Hachihonmatsu, Higashihiroshima. Hiroshima 739–0151, Japan; 4 Program of Biotechnology, Graduate School of Integrated Sciences for Life, Hiroshima University, 1–3–1, Kagamiyama, Higashi-Hiroshima, Hiroshima 739–8530, Japan; 5 Department of Civil and Environmental Engineering, Graduate School of Advanced Science and Engineering, Hiroshima University, 1–4–1, Kagamiyama, Higashi-Hiroshima, Hiroshima 739–8527, Japan

**Keywords:** methane-oxidizing bacteria, environmental factors, pH, diversity, *Mycobacterium*

## Abstract

Methane-oxidizing bacteria (MOB) are ubiquitous and play an important role in the mitigation of global warming by reducing methane. MOB are commonly classified into Type I and Type II, belonging to *Gammaproteobacteria* and *Alphaproteobacteria*, respectively, and the diversity of MOB has been examined. However, limited information is currently available on favorable environments for the respective MOB. To investigate the environmental factors affecting the dominant type in the MOB community, we performed MOB enrichment using down-flow hanging sponge reactors under 38 different environmental conditions with a wide range of methane (0.01–80%) and ammonium concentrations (0.001–2,000‍ ‍mg N L^–1^) and pH 4–7. Enrichment results revealed that pH was a crucial factor influencing the MOB type enriched. Type II was dominantly enriched at low pH (4–5), whereas Type I was dominant around neutral pH (6–7). However, there were some unusual cultivated biomass samples. Even though high methane oxidation activity was observed, very few or zero conventional MOB were detected using common FISH probes and primer sets for the 16S rRNA gene and *pmoA* gene amplification. *Mycobacterium* mostly dominated the microbial community in the biomass cultivated at very high NH_4_^+^ concentrations, strongly implying that it exhibits methane oxidation activity. Collectively, the present results revealed the presence of many unknown phylogenetic groups with the capacity for methane oxidation other than the reported MOB.

Methane is the second largest contributor to global warming, and its atmospheric concentration has been rapidly increasing, reaching approximately 1,850 ppb in 2017 (Nisbet *et al.*, 2019). Although methane is biologically produced in nature, methane-oxidizing bacteria (MOB) play an important role in reducing its atmospheric concentration, which contributes to the mitigation of global warming (Conrad, 2009). MOB are commonly classified into two types, *i.e*., Type I and Type II, based on phylogenetic and metabolic differences (Hanson and Hanson, 1996). Type I MOB belong to *Gammaproteobacteria* that use the ribulose monophosphate (RuMP) pathway for carbon assimilation, while Type II MOB belong to *Alphaproteobacteria* that use the serine pathway. Besides *Proteobacteria*, recent studies reported new groups of MOB, such as *Methylacidiphilum fumariolicum* in the phylum *Verrucomicrobia* (Pol *et al.*, 2007). Ettwig *et al.* (2009) found the NC10 phylum *Candidatus Methylomirabilis oxygeniifera*. Therefore, methane is consumed by diverse MOB. However, limited information is currently available on the specific environmental conditions required for different MOB and the extent to which they contribute to the carbon cycle.

Since pH affects microbial activity, the type of dominant MOB in an environment depends on its pH. Type II MOB are often detected in acidic environments, such as forest soils and peat (Dedysh *et al.*, 2001; 2003). In contrast, Type I are found under neutral pH and alkaline conditions (Khmelenina *et al.*, 2010; Gupta *et al.*, 2012), suggesting that pH is an important factor governing the MOB community structure. However, different pH dependencies have been reported during the enrichment of the MOB community in bioreactors. Hatamoto *et al.* (2011) reported that Type II MOB dominated not only at pH 5.5, but also at pH 8.0.

In addition to pH, the concentration of methane may also affect the MOB community structure. If two MOB types have different affinities for methane, the dominant type will depend on the concentration of methane present. Type I MOB reportedly exhibit a preference for lower methane concentrations than Type II, which preferentially grow at higher methane concentrations (Graham *et al.*, 1993; Amaral and Knowles, 1995). However, Matsuura *et al.* (2017) obtained different findings in a reactor with a range of methane concentrations, with only Type I MOB dominating at both high and low concentrations.

Field surveys of farm soils demonstrated that ammonium fertilizers reduced methane oxidation (Steudler *et al.*, 1989; Mohanty *et al.*, 2006; Shrestha *et al.*, 2010). This phenomenon may be explained by MOB simultaneously oxidizing ammonium to nitrite (Hutton and Zobell, 1953; Dalton, 1977; O’Neill and Wilkinson, 1977). MOB exhibit methane monooxygenase activity, which oxidizes methane to methanol. Ammonium, which has a similar molecular structure and size to methane, binds to the active site of methane monooxygenase and is oxidized (Schimel, 2000). In other words, ammonium inhibits methane oxidation, and this competitive inhibition has been observed for different MOB species in pure culture experiments (Whittenbury *et al.*, 1970; King and Schnell, 1994). In addition, the degree of inhibition differs between various species of MOB (Nyerges and Stein, 2009; Nyerges *et al.*, 2010). Therefore, the concentration of ammonium in an environment may also influence the dominance of a particular MOB.

Methanotrophic community ana­lyses of soil samples from a wide variety of environments, such as farm paddy fields and forests, have been conducted (*e.g.*, Bourne *et al.*, 2001; Knief *et al.*, 2005; Lüke and Frenzel, 2011; Kravchenko and Sukhacheva, 2017). This type of survey is very useful for obtaining a more detailed understanding of the diversity of MOB in natural environments. However, the methodology employed has a significant drawback for assessing MOB-specific growth conditions because of the difficulties associated with measuring methane and ammonium concentrations in natural environments. In contrast, the use of bioreactors with a continuous flow operation is very promising for examining the relationship between habitant MOB and environments because experimental conditions may be easily controlled and properly maintained for a long time during MOB enrichment. Moreover, MOB produce intermediates (Hanson and Hanson, 1996) that affect experimental conditions; however, these may be easily discharged from continuous bioreactors.

To identify the environmental factors governing the MOB community structure, we attempted to enrich diverse MOB under different environmental conditions of pH, methane concentrations, and ammonium concentrations. As one of the features of the present study, a down-flow hanging sponge (DHS) reactor was employed because it has been successfully implemented in MOB cultivation (Hatamoto *et al.*, 2011; Matsuura *et al.*, 2017; Matsushita *et al.*, 2018) and also in the enrichment of unculturable microbes (Imachi *et al.*, 2020), and it is easy to operate.

## Materials and Methods

### Experimental set-up

To enrich MOB, 38 identical closed DHS reactors (each consisting of a 70-mL glass column with a height of 400‍ ‍mm and diameter of 30‍ ‍mm) were used. A set of eight polyurethane sponge cubes (each 1×1×1‍ ‍cm^3^) were hung diagonally in series on a nylon string in the gas phase of each column. Activated sludge collected from an aeration tank in a municipal wastewater treatment plant was used as the microbial inoculum. Before setting up the DHS reactors, sponges were soaked in activated sludge diluted with water (approximately 100‍ ‍mg MLSS L^–1^).

### Operational conditions

All reactors were operated in a temperature-controlled dark room at 30°C for 13–89 days, depending on methane consumption (Table 1). Air containing methane as the substrate was fed into the top of each reactor at a gas retention time (GRT) of 0.3–14.5‍ ‍min based on the sponge volume using a pump and emitted from the bottom of the column. A mineral solution was supplied to the reactor at a hydraulic retention time (HRT) of 0.4–87‍ ‍min based on the sponge volume and discharged from the bottom. Effluents were recirculated at a ratio of 1:10 (influent : recirculation) to mitigate the longitudinal methane concentration gradient in the liquid and gas. Feeding methane concentrations were set at 0.01, 0.1, 1.0, 5.0, 10, and 80%. Influent ammonium was provided at 0.001, 0.1, 0.5, 1.0, 10, 100, 500, 1,000, and 2,000‍ ‍mg NH_4_^+^ N L^–1^. Influent pH was set at 4, 5, 6, and 7. Oxygen was provided at 20%, except for Runs 3 and 7 (for which it was provided at 2.0%). Thirty-eight runs were conducted using different combinations of methane concentrations, ammonium concentrations, oxygen concentrations, and pH values (Table 1).

### Mineral composition

The mineral solution comprised 5‍ ‍mg L^–1^ CaCl_2_·2H_2_O, 33‍ ‍mg L^–1^ MgCl_2_·6H_2_O; 16‍ ‍mg L^–1^ KCl; 7.2‍ ‍mg L^–1^ KNO_3_; 5.49‍ ‍mg L^–1^ FeSO_4_·7H_2_O; 0.17‍ ‍mg L^–1^ CoCl_2_·6H_2_O; 0.15‍ ‍mg L^–1^ ZnSO_4_·7H_2_O; 0.06‍ ‍mg L^–1^ H_3_BO_3_; 0.04‍ ‍mg L^–1^ MnCl_2_·4H_2_O; 0.027‍ ‍mg L^–1^ CuCl_2_·2H_2_O; 0.025mg L^–1^ Na_2_MoO_4_·2H_2_O; 0.013‍ ‍mg L^–1^ AlCl_3_; 0.024‍ ‍mg L^–1^ NiCl_2_ 6H_2_O; 0.0017‍ ‍mg L^–1^ Na_2_SeO_4_; 0.0033‍ ‍mg‍ ‍L^–1^ Na_2_WO_4_·2H_2_O. Ammonium was added using NH_4_Cl. pH was adjusted using KH_2_PO_4_ (8.3–82.9‍ ‍mg L^–1^), Na_2_HPO_4_ (197.5–19.8‍ ‍mg L^–1^), and H_2_SO_4_.

### Methane oxidation activity test

After MOB were enriched and methane oxidation rates had almost reached a steady state, methane oxidation activity tests were conducted on four biomass samples from Runs 29, 30, 31, and 32 to investigate the effects of the concentration of NH_4_^+^ and pH on MOB activity. Four samples, in which Type I or Type II MOB were predicted to be dominantly enriched, were selected as representatives of Type I and Type II MOB. Activity tests were performed using DHS reactors at methane concentrations of 10 and 0.1% directly after MOB enrichment. The concentrations of NH_4_^+^ in the test liquid were 0.1, 1.0, 10, 100, and 1,000‍ ‍mg N L^–1^, and pH was set at 4, 5, 6, and 7. Therefore, 40 different conditions were applied for each biomass. Methane oxidation activity was evaluated from methane concentrations in the influent gas and off-gas, and GRT. The duration of tests was shortened as much as possible to prevent a microbial community change during activity testing. Five tests at the different NH_4_^+^ concentrations were conducted in 1 day. Moreover, on the next day, each DHS reactor was operated under the original enrichment condition, and the recovery of methane oxidation activity was assessed.

### Analytical methods

The supply gas and off-gas were stored and collected in a gas bag (GL Science Aluminum bag), and methane concentrations were measured using a gas chromatograph equipped with a thermal conductivity detector (GC-8A; Shimadzu) for high concentration samples (Dinh *et al.*, 2021) and a gas chromatograph equipped with a flame ionization detector (GC-2014; Shimadzu) for low concentration samples. The concentration of NH_4_^+^ was measured by Nessler’s method using a Hach spectrophotometer (DR2800; Hach) according to the manufacturer’s instructions. pH was measured using a pH meter.

### Biomass sampling and microbial community ana­lysis

Biomass sampling was conducted by squeezing the sponge carriers at the end of the operation, as previously described, to perform a microbial community ana­lysis (Matsushita *et al.*, 2020).

### Fluorescence *in situ* hybridization (FISH)

During the FISH ana­lysis, sample fixation and hybridization were conducted according to the procedure described by Awata *et al.* (2013). The EUB338, EUB338-II, and EUB338-III probes (Daims *et al.*, 1999) labeled with Alexa Fluor 647 were used to evaluate all bacteria. Probes of Mγ84 and Mγ705 specific for Type I MOB and Mα450 detecting Type II MOB (Eller *et al.*, 2001) were labeled with Cy3 or Alexa 488 depending on the combination of the probes used. Hybridized samples were observed using an epifluorescence microscope (Axio Imager M1; Carl Zeiss).

### 16S rRNA gene ana­lysis

The collected biomass was washed with phosphate buffer. DNA was extracted using the FastDNA^®^ SPIN Kit for Soil (MP Biomedicals) according to the manufacturer’s instructions.

PCR amplification of the 16S rRNA gene was conducted using the primer set containing 341' f (5′-CCTACGGGNGGCWGCAG-3′) and 805r (5′-GACTACHVGGGTATCTAATCC-3′) for the V3V4 regions, using One Shot LA PCR Mix Ver. 2.0 (Takara Bio). PCR was performed with an initial denaturation at 95°C for 5‍ ‍min, followed by 30 cycles at 95°C for 30‍ ‍s, at 58°C for 30‍ ‍s, and at 72°C for 30 s; final extension was performed at 72°C for 5‍ ‍min. PCR products were purified using Agencourt AMPure XP (Beckman Coulter) and sequenced using Roche GS Junior (454 Life Sciences) at Hokkaido System Science.

Regarding samples with insufficient PCR products, second PCR was performed with TaKaRa Ex Taq^®️^ Hot Start Version (Takara Bio). Reaction conditions were as follows: initial denaturation at 94°C for 2‍ ‍min, followed by 30 cycles at 94°C for 30‍ ‍s, at 50°C for 30‍ ‍s, and at 72°C for 30 s; final extension was performed at 72°C for 5‍ ‍min. PCR products were purified using the same procedure and sequenced using the Illumina MiSeq platform with MiSeq reagent kit v3 (Illumina) at Bioengineering Lab.

To specifically amplify the 16S rRNA gene of Type I MOB, the FISH probes of Mγ84f and Mγ705r specific for Type I were used as primers. Using two newly designed primer sets of Mγ84f (5′-TCGGGCGCTGACGAGTGG-3′)/341'r (5′-CTGCWGCCNCCCGTAGG-3′) and 341 ' f (5′-CCTACGGGNGGCWGCAG-3′)/Mγ705r (5′-CTGGTGTTCCTTCAGATC-3′), PCR was performed on DNA samples from Runs 1 and 5 using TaKaRa Ex Taq^®️^ Hot Start Version by an initial denaturation at 94°C for 2‍ ‍min, followed by 30 cycles at 94°C for 30‍ ‍s, at 59 or 50°C for 30‍ ‍s, and at 72°C for 30 s; final extension was performed at 72°C for 5‍ ‍min. PCR products were purified and sequenced using the Illumina MiSeq platform with the MiSeq reagent kit v3 at Bioengineering Lab.

### Particulate methane monooxygenase gene *pmoA* ana­lysis

The *pmoA* gene was amplified using the primer set of A189f (5′-GGNGACTGGGACTTCTGG-3′) and mb661r (5′-CCGGMGCAACGTCYTTACC-3′) (McDonald *et al.*, 2008). PCR was performed using One Shot LA PCR Mix Ver. 2.0 by an initial denaturation at 95°C for 5‍ ‍min, followed by 25–45 cycles at 95°C for 30‍ ‍s, at 50°C for 30‍ ‍s, and at 72°C for 1‍ ‍min; final extension was performed at 72°C for 5‍ ‍min. PCR products were sequenced using the Illumina MiSeq platform with MiSeq reagent kit v3 at Kanazawa University.

### Phylogenetic ana­lysis

Raw sequence data were trimmed using Cutadapt software (version 1.18) (Parada *et al.*, 2016) to remove primers from the sequence reads, as previously described (Dinh *et al.*, 2022). Clean reads were analyzed using QIIME 2 Core 2020.8 (Bolyen *et al.*, 2019). Operational taxonomic units (OTUs)were grouped using the pipeline software DADA2 (Callahan *et al.*, 2016) with the SILVA (release_132) database (Pruesse *et al.*, 2012; Quast *et al.*, 2013; Yilmaz *et al.*, 2014) and *pmoA* gene database (Dumont *et al.*, 2014). Sequence data were deposited in the DNA Data Bank of Japan (DDBJ) database under DDBJ/EMBL/GenBank accession number DRA012996.

## Results

### Time course of methane oxidation and enrichment of MOB

**Fig. 1.**
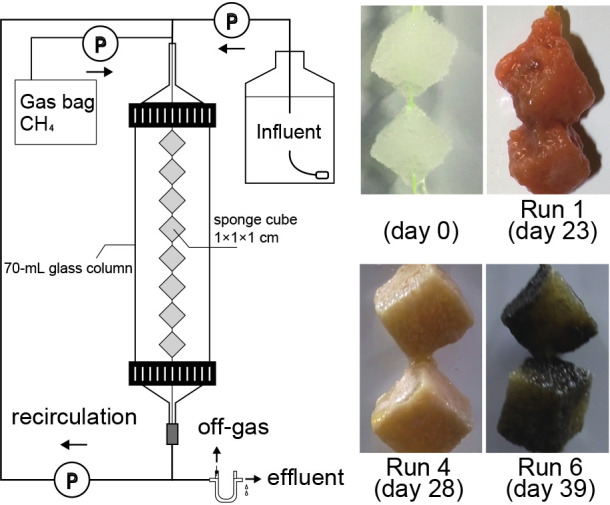
Schematic diagram of the closed DHS reactor and photographs of representative biomass samples cultivated on sponges in three runs.

MOB were successfully enriched for all 38 runs operated under different conditions, as shown in Table 1. The respective methane oxidation rates based on the sponge volume (R_m_) increased with time and almost reached a stationary state at the end of the cultivation period (Fig. S1). However, the time required to achieve a plateau and the maximum R_m_ at that time markedly differed among the 38 runs (Table 1). The biomass gradually growing on the sponges was visually observed with increasing R_m_. Biomass colors varied across the runs and included orange, white, and black (Fig. 1 and S3).

**Fig. 2.**
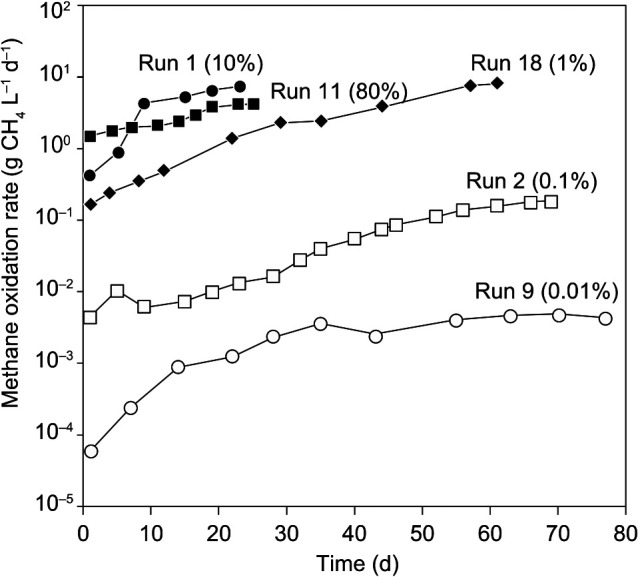
Time courses of methane oxidation rates in five representative runs. Values in parentheses indicate methane concentrations in the gas provided.

Fig. 2 shows the time course of R_m_ for five runs as an example. Run 1, operated at a high methane concentration of 10%, had a high R_m_ with a maximum of 7.37‍ ‍g CH_4_ L^–1^‍ ‍d^–1^, even for a short operation period. In contrast, R_m_ in Run 9 at a very low methane concentration of 0.01% exhibited a very low R_m_ with a maximum of 0.004‍ ‍g CH_4_ L^–1^ d^–1^, and the operation period had to be prolonged to 77 days for the enrichment of MOB with sufficient biomass sampling. R_m_ in the stationary state slightly increased with higher methane concentrations up to 10% (Fig. S2). Since a higher methane concentration (80%) may slightly inhibit MOB activity, the enrichment of MOB was performed even under conditions of very low methane concentrations; however, longer operations were necessary.

As methane was consumed in the reactor, a difference was observed between the provided gas and off-gas. We carefully increased the methane loading rate during the operation of the reactor by gradually reducing GRT in a manner than depended on the amount of methane consumed in order to maintain a small difference. Therefore, the off-gas concentration for all runs was maintained within 65–95% of the provided gas concentration (Table S1). Regarding the liquid, a short HRT was also maintained to keep the pH and NH_4_^+^ concentration constant in the reactor, thereby retaining these values in the effluent. A vertical concentration gradient did not occur for the liquid or gas in the reactor because high recirculation was performed, implying that methane concentrations in the reactor were the same as those in the effluent and off-gas. Therefore, the enrichment of MOB was successfully conducted as planned under different environmental conditions.

### The dominant MOB type identified by FISH

**Fig. 3.**
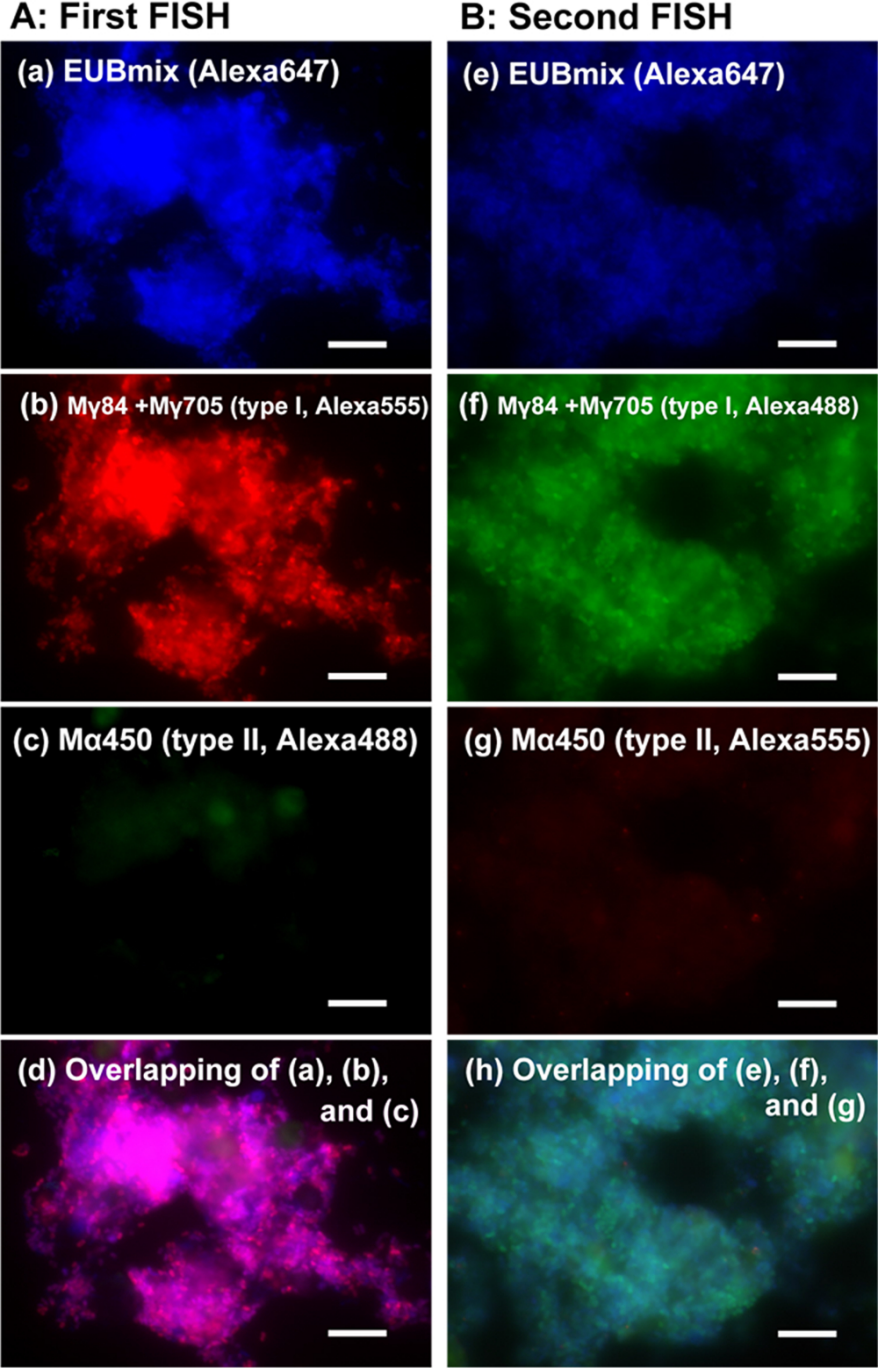
Fluorescence images from the FISH ana­lysis for the biomass from Run 1 with two different combinations of labels. (A) The first FISH using EUB mix probes labeled with Alexa 647 (blue), Mγ84 and Mγ705 probes labeled with Alexa 555 (red) to detect type I MOB, and the Mα450 probe labeled with Alexa 488 (green) to detect type II. (B) The second FISH using EUB mix probes labeled with Alexa 647 (blue), Mγ84 and Mγ705 probes labeled with Alexa 488 (green), and the Mα450 probe labeled with Alexa 555 (red). Scale bars represent 10‍ ‍μm.

FISH was performed to investigate whether the dominant MOB enriched belonged to Type I or Type II. Two probes, Mγ84 and Mγ705, specific for Type I, and the probe Mα450 specific for Type II were used with the mixed universal eubacterial probes of EUB 338 (referred to as EUB mix) labeled with Alexa 647 (blue). A combination of Type I-targeting probes labeled with Alexa 555 (red) and a Type II-targeting probe labeled with Alexa 488 (green) was applied. In the biomass sample from Run 1, the dominant MOB were Type I because fluorescence from Type I and EUB mix was detected from the same cells, as shown in Fig. 3A. However, difficulties were associated with identifying some samples as Type I or II because the multi-pseudocolor of fluorescence was occasionally unclear. Therefore, to confirm the type, a second FISH ana­lysis was conducted at different fluorescence combinations, where Alexa 555 and Alexa 488 were exchanged. After the second FISH, the dominant MOB from Run 1 were classified as Type I (Fig. 3B). Therefore, we confirmed that Type I MOB were enriched in Run 1 based on the results of two FISH assays. Similarly, the dominant MOB type was identified in 25 samples, as shown in Table 1 and Fig. S4. However, it was challenging to identify the dominant MOB in the remaining 13 samples for the following reasons: 1) fluorescence from MOB was weak, and 2) three fluorescence bands from EUB mix, Type I, and Type II probes were simultaneously detected from the same fields (Table 1 and Fig. S4), which are discussed in more detail below.

### Environmental conditions affecting the type of enriched MOB

**Fig. 4.**
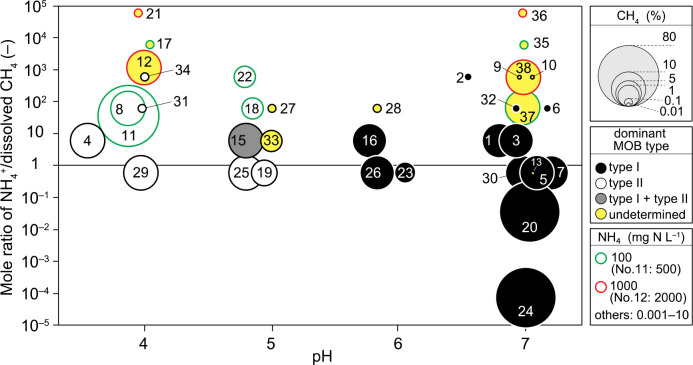
Relationship between the dominant MOB type identified by FISH and pH, CH_4_ concentrations, and the mole ratio of NH_4_/dissolved CH_4_. The numeral beside the circle represents the run number.

The enrichment of MOB was conducted under a wide range of conditions: pH 4–7; methane 0.01–80%; NH_4_^+^ 0.001–2,000‍ ‍mg‍ ‍N‍ ‍L^–1^. These three environmental factors influence the MOB type enriched. To identify the crucial factors influencing the MOB type, we created a unique and at-a-glance figure to recognize the complex relationships among the type, pH, methane concentration, NH_4_^+^ concentration, and mole ratio of NH_4_^+^ to dissolved methane (NH_4_^+^/CH_4_), as shown in Fig. 4. The dominant MOB type was separated depending on pH, except for unjudged runs, suggesting that pH was the crucial factor governing the MOB type. Type II dominated at low pH (4–5), while Type I was dominant at near-neutral pH (6–7). Both types were detected in Run 15 enriched at pH 5, plotted in the vicinity of the boundary splitting the dominant types.

Regarding runs in which the dominant MOB type was not identified, some characteristics were found in the operational conditions. Among 13 of these samples, 12 were enriched under the conditions of a higher NH_4_^+^/CH_4_ ratio with very high NH_4_^+^ concentrations. The effect of the high NH_4_^+^/CH_4_ ratio was significant at pH 7, at which the identification of the MOB type was not performed at a ratio of 10. However, even though the NH_4_^+^/CH_4_ ratio was very high, identification was possible for Runs 2, 6, and 32 when NH_4_^+^ concentrations were not high. In addition, even when the CH_4_ concentration was very low (0.01%) in Runs 9, 10, and 13, it was impossible to identify the dominant type. These results suggest that unknown MOB, undetectable by a FISH ana­lysis, were cultivated under the conditions of very high NH_4_^+^ concentrations and very low CH_4_ concentrations. Therefore, although pH was the crucial factor governing the enriched MOB type, NH_4_^+^ and CH_4_ concentrations were also important.

### Effects of pH and NH_4_^+^ on the methane oxidation rate, R_m_

As described above, the type of enriched MOB was clearly dependent on the pH condition. If the optimum pH of MOB activity differs between Types I and II, the reason the enriched type was separated at pH 5–6 may be explained. Therefore, the respective methane oxidation rates of Types I and II were investigated under widely different pH conditions. Four biomass samples from Runs 29, 30, 31, and 32 were employed for this investigation because the dominant types in these runs were clearly identified using FISH. The representatives of Type I samples were Runs 30 and 32, which were conducted at pH 7 with methane concentrations of 10 and 0.1%, respectively. In contrast, for Type II, samples from Runs 29 and 31 were used, which were enriched at pH 4 with methane concentrations of 10 and 0.1%, respectively.

**Fig. 5.**
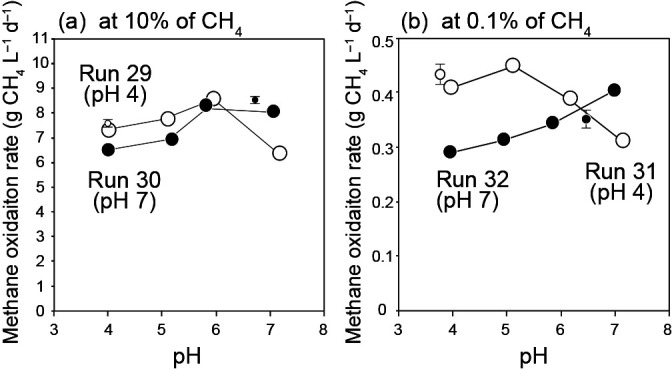
Effects of pH on the methane oxidation rate at (a) a methane concentration of 10% for biomass samples from Runs 29 and 30, and (b) a methane concentration of 0.1% for samples from Runs 31 and 32. pH in the parentheses indicates the original pH value during the enrichment.

Fig. 5 shows the influence of pH on the methane oxidation rate, R_m_ at the two different methane concentrations of 10 and 0.1% with a low NH_4_^+^ concentration of 0.1‍ ‍mg‍ ‍N‍ ‍L^–1^, applied to prevent NH_4_^+^-mediated inhibition. No significant differences were observed between the R_m_ values of the Type I biomass from Run 30 and the Type II biomass from Run 29 (Fig. 5a). However, a marked difference was noted under the influence of pH. In the Type I biomass, R_m_ slightly declined with a decrease in pH, with the highest value being obtained at pH 7. In contrast, R_m_ of the Type II biomass had an optimum pH of 6 and markedly decreased at pH 7. In other words, the R_m_ values of Type I and Type II biomass samples crossed at pH 4–7 under a methane concentration of 10%. This influence of pH on R_m_ was also observed for the biomass samples from Runs 31 and 32, which were conducted using a methane concentration of 0.1% (Fig. 5b). Even at significantly different methane concentrations of 0.1 and 10%, the same phenomenon was observed for the influence of pH, suggesting that the affinities of Types I and II MOB for CH_4_ were similar. Consequently, Type I MOB appear to be preferentially enriched under neutral pH conditions, and Type II under acidic conditions, which strongly supports the results of the FISH ana­lysis and may be explained by the characteristics of Type I and Type II MOB.

**Fig. 6.**
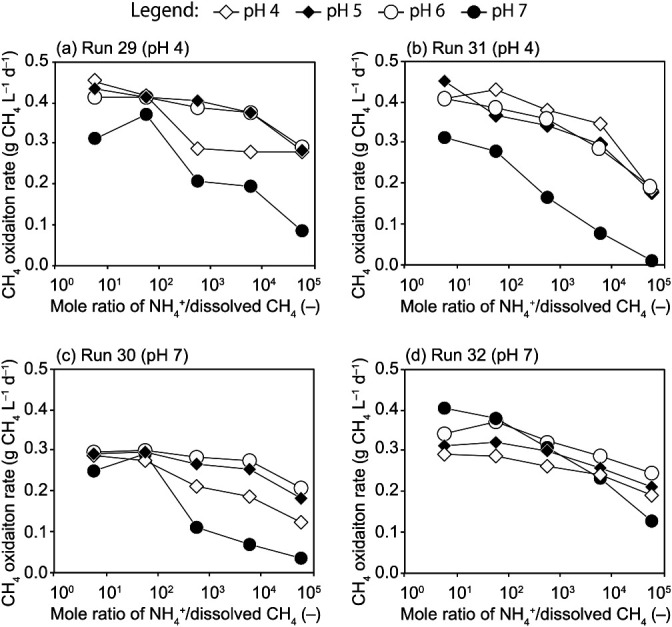
Effects of the mole ratio of NH_4_^+^/dissolved CH_4_ on the methane oxidation rate at 0.1% CH_4_ under different pH conditions for samples from Runs 29 to 32. pH in the parentheses indicates the original pH value during the enrichment.

The inhibition of ammonium was observed in the cultivation of MOB because R_m_ at the stationary stage appeared to decrease with increasing concentrations of NH_4_^+^ (Fig. S2). If the intensity of the inhibition of ammonium markedly differs between Types I and II, it will influence the dominant type. Therefore, the influence of NH_4_^+^ concentrations on R_m_ was also investigated using four biomass samples from Runs 29, 30, 31, and 32. R_m_ was estimated under the conditions of pH 4–7, NH_4_^+^ concentrations of 0.1–1,000‍ ‍mg N L^–1^, and a methane concentration of 0.1%. Since the inhibition of ammonium is competitive with methane, the mole ratio of NH_4_^+^/dissolved CH_4_ was applied to evaluate the effects of inhibition. Fig. 6 shows R_m_ versus the NH_4_^+^/CH_4_ ratio. R_m_ steadily decreased with an increase in the NH_4_^+^/CH_4_ ratio in all four biomass samples at any pH. Moreover, the degradation of R_m_ was marked at pH 7, with a decrease of approximately 30–95% from the maximum value. This negative effect of the NH_4_^+^/CH_4_ ratio was also detected while investigating the influence of a high methane concentration (10%) on R_m_ (Fig. S5). High NH_4_^+^ concentrations were found to inhibit the activities of both Type I and Type II MOB, and the intensity of inhibition was similar at any pH.

Differences in affinity for CH_4_ and the intensity of NH_4_^+^ inhibition were small between Type I and Type II MOB. In contrast, the optimum pH of R_m_ markedly differed. Therefore, the enriched MOB type may only be governed by pH conditions, even though NH_4_^+^ concentrations had a significant negative impact on MOB activity.

### Microbial ana­lysis

**Fig. 7.**
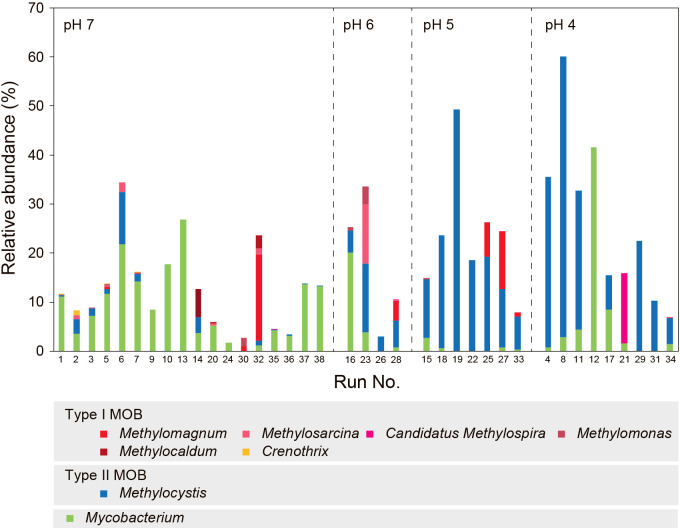
Relative abundance of MOB and *Mycobacterium* based on 16S rRNA genes.

In addition to the FISH ana­lysis, other microbial community ana­lyses were performed to obtain more detailed information on the microbial population structures based on 16S rRNA and the functional *pmoA* gene using the primer sets 341'f/805r and A189f/mb661r, respectively. The number of reads is shown in Table S3, and the results of microbial ana­lyses in Fig. 7, S6, S7, and S8. The dominant MOB were estimated to be Type II for the majority of samples (Fig. 7, S7, and S8), which was completely different from the results of the FISH ana­lysis. Even though MOB in Runs 1 and 5 were classified as Type I by the FISH ana­lysis, they were estimated to be Type II by both *pmoA* and 16S rRNA gene ana­lyses. We speculated that the detection of Type I MOB may be difficult by PCR with the primer sets used for some samples.

Therefore, we also conducted a 16S rRNA gene ana­lysis using two newly designed primer sets, Mγ84f/341'r and 341'f/Mγ705r, in which the sequences of Mγ84 and Mγ705 were used as FISH probes targeting Type I, and applied to the samples of Runs 1 and 5. The results obtained showed that *Methylomonas* and *Methylomagnum* of MOB Type I were dominant, as expected (Table S2). Consequently, the results of *pmoA* and 16S rRNA gene ana­lyses may have a large bias derived from PCR amplification depending on the primer sets.

## Discussion

The FISH ana­lysis conducted in the present study revealed that pH was a crucial factor governing the MOB type enriched, with Type II appearing at low pH of 4–5 and Type I being dominant at neutral pH of 6–7. The present results on the effects of pH on the MOB type are consistent with previous findings. Dedysh *et al.* (2001; 2003) reported that the dominant MOB under acidic pH, such as in forests and peats, were Type II, with Type I accounting for less than 1%. Type I MOB were reportedly detected at neutral pH of 6.5–7.5 (Shrestha *et al.*, 2010; Gupta *et al.*, 2012). Type I and Type II MOB were both shown to be enriched around the pH boundary that separates the two types of MOB. Hatamoto *et al.* (2011) found that the dominant MOB were Type II in a DHS reactor, cultivated at pH 5.5 under a low methane concentration. Even at a similar pH of 5.6, Noorain *et al.* (2019) showed that Type I MOB were dominantly cultivated in a DHS reactor at a high methane concentration. At a pH of approximately 5.5, the dominant MOB type was unclear because of the very small difference in the methane consumption rates of Type I and Type II MOB with weak pH dependency, as shown in Fig. 5.

However, the findings of other studies contradict the present results. Even at neutral pH, Type I and Type II MOB have both been detected (*e.g.* Hatamoto *et al.*, 2010; Pfluger *et al.*, 2011; Matsuura *et al.*, 2017), with the identification of the MOB type being based on the *pmoA* gene using the primer set of A189f/mb661r. Although Bourne *et al.* (2001) reported that the primer set of A189f/mb661r was superior to A189f/A682r and A189f/A650r for the wide detection of diverse MOB, Cai *et al.* (2020) indicated that *Methylocystis* of Type II MOB may be preferentially detected in a microbial ana­lysis using A189f/mb661r. These findings suggest that the results of microbial ana­lyses are strongly dependent on the selection of primer sets. In the present study, the effects of pH dependency were derived from a FISH ana­lysis. Therefore, the discrepancy between the present results and previous findings on dominant MOB may be attributed to the different methods used for ana­lyses.

Ammonium inhibits MOB activity (Whittenbury *et al.*, 1970; Carlsen *et al.*, 1991; Dunfield and Knowles, 1995) because its molecular structure is very similar to that of methane, resulting in the competitive inhibition of methane monooxygenase. The inhibition of methane oxidation due to ammonium was also observed in the present study. Therefore, we hypothesized that ammonium may affect the type of MOB enriched. Mohanty *et al.* (2006) reported that ammonium stimulated methane consumption by Type I MOB in rice field soils, while the enrichment of Type II MOB was inhibited. However, the present results showed that ammonium did not affect the enriched MOB type over a wide range of concentrations because the methane oxidation rates of Type I (Runs 30 and 32) and Type II (Runs 29 and 31) MOB were similarly affected by the mole ratio of NH_4_^+^/dissolved CH_4_ (Fig. 6 and S5).

Although methane consumption was observed even for runs even under a very low methane concentration (0.01%=100 ppm), high NH_4_^+^/CH_4_ ratio, and very high NH_4_^+^ concentrations of more than 2,000‍ ‍mg N L^–1^, MOB in these biomass samples were undetectable by FISH using the probes Mγ84, Mγ705, and Mα450 (Table 1). Therefore, unknown MOB that cannot be detected using conventional probes may exist.

Regarding MOB utilizing methane even at low concentrations, *Methylocystis* sp. strain SC2 was shown to exhibit activity in a methane concentration range of 1.75 to 100‍ ‍ppm, with two isozymes of methane monooxygenase with different methane oxidation kinetics, *pmoA1*, which has low affinity for methane, and *pmoA2*, which has high affinity (Baani and Liesack, 2008). Previous studies reported a role for the *pmoA2* gene in atmospheric methane consumption in forests (Kravchenko *et al.*, 2010) and paddy soils (Cai *et al.*, 2016). In addition, uncultivated MOB affiliated with the USCα and USCγ groups were shown to be involved in the oxidation of atmospheric methane (Kolb, 2009). Tveit *et al.* (2019) successfully isolated *Methylocapsa gorgona* MG08 belonging to USCα, the first MOB capable of not only consuming atmospheric methane, but also growing on it. These findings and the present results suggest that the diversity of MOB is also derived from various affinities for methane.

Ammonia-oxidizing bacteria (AOB) were detected in several runs, specifically at pH 7 (Fig. S7). AOB slightly oxidize methane because ammonia monooxygenase, a key enzyme in nitrification, is phylogenetically very close to methane monooxygenase (Jones and Morita, 1983; Bédard and Knowles, 1989). We considered the possibility of methane removal by AOB in runs performed under high NH_4_^+^/CH_4_ conditions at least once. However, the methane oxidation activity of AOB is generally very low (Bédard and Knowles, 1989), and R_m_ at a high NH_4_/CH_4_ ratio was similar to that at other ratios (Table 1). Furthermore, AOB were not detected at pH 4 because their enrichment is typically impossible in a highly acidic environment.

In the runs performed at very high NH_4_^+^ concentrations corresponding to a high NH_4_/CH_4_ ratio, *Mycobacterium* was dominant. The abundance of *Mycobacterium* was 41.6% in Run 12 enriched at an NH_4_^+^ concentration of 2,000‍ ‍mg N L^–1^ from the 16S rRNA gene ana­lysis, while no MOB were detected, even in the FISH ana­lysis. Reed and Dugan (1987) initially reported that *Mycobacterium* ID-Y oxidized methane. However, to the best of our knowledge, there is currently no evidence to support methane oxidation by *Mycobacterium*. *Mycobacterium chubuense* stain NBB4 was shown to metabolize the alkanes and alkenes of C_2_ to C_4_ using a soluble methane monooxygenase-like enzyme (Martin *et al.*, 2014). Since this enzyme is phylogenetically close to the conventional methane monooxygenase, some species possessing specific types of the enzyme may oxidize methane; however, only Reed and Dugan (1987) reported methane oxidation by *Mycobacterium*.

Since methane provided in the present experiments was the sole organic carbon source, bacteria highly enriched with a dominance of more than 40% appeared to utilize methane, indicating that the enriched *Mycobacterium* is a methanotroph. Furthermore, the *Mycobacterium* detected grew even at very high NH_4_^+^ concentrations, at which common MOB are inhibited. Further studies are needed to establish whether the new MOB implied by the present results exist in the genus *Mycobacterium*.

## Citation

Kambara, H., Shinno, T., Matsuura, N., Matsushita, S., Aoi, Y., Kindaichi, T., et al. (2022) Environmental Factors Affecting the Community of Methane-oxidizing Bacteria. *Microbes Environ ***37**: ME21074.

https://doi.org/10.1264/jsme2.ME21074

## Supplementary Material

Supplementary Material

## Figures and Tables

**Table 1.** Operational conditions, maximum methane oxidation rates, and dominant MOB types identified by FISH

Run No.	pH	CH_4_ (%)	NH_4_^+^ (mg N L^–1^)	O_2_ (%)	Cultivation period (day)	CH_4_ oxidation rate (g CH_4_ L^–1^ d^–1^)	Dominant MOB type
1	7	10	10	20	23	7.367	Type I
2	7	0.1	10	20	69	0.187	Type I
3	7	10	10	2	35	3.848	Type I
4	4	10	10	20	28	6.795	Type II
5	7	10	1.0	20	13	9.985	Type I
6	7	0.1	1.0	20	39	0.238	Type I
7	7	10	1.0	2	13	6.784	Type I
8	4	10	100	20	18	9.843	Type II
9	7	0.01	1.0	20	77	0.004	ND^1^
10	7	0.01	1.0	20	77	0.001	ND^1^
11	4	80	500	20	25	4.228	Type II
12	4	10	2000	20	98	4.117	ND^1^
13	7	0.01	0.001	20	85	0.004	ND^1^
14	7	0.1	0.001	20	83	0.219	Type I
15	5	10	10	20	33	8.500	Type I+II
16	6	10	10	20	17	8.008	Type I
17	4	0.1	100	20	57	0.363	ND^2^
18	5	1.0	10	20	61	8.029	Type II
19	5	5.0	0.5	20	29	5.232	Type II
20	7	80	0.5	20	17	3.328	Type I
21	4	0.1	1000	20	83	0.252	ND^1^
22	5	1.0	100	20	37	4.104	Type II
23	6	1.0	0.1	20	37	2.367	Type I
24	7	80	0.001	20	17	1.313	Type I
25	5	10	1.0	20	14	8.307	Type II
26	6	10	1.0	20	14	7.640	Type I
27	5	0.1	1.0	20	89	0.683	ND^1^
28	6	0.1	1.0	20	89	0.566	ND^1^
29	4	10	1.0	20	22	6.577	Type II
30	7	10	1.0	20	22	8.022	Type I
31	4	0.1	1.0	20	73	0.509	Type II
32	7	0.1	1.0	20	73	0.453	Type I
33	5	1.0	1.0	20	44	2.626	ND^1^
34	4	0.1	10	20	44	0.210	Type II
35	7	0.1	100	20	44	0.052	ND^2^
36	7	0.1	1000	20	44	0.019	ND^1^
37	7	10	100	20	19	5.263	ND^1^
38	7	10	1000	20	19	3.326	ND^1^

ND^1^: not determined because of weak fluorescence. ND^2^: not determined because three fluorescence bands from the EUB mix, Type I, and Type II probes were simultaneously observed from the same cells.

## Abbreviations

Ammonia-oxidizing bacteria (AOB)

Down-flow hanging sponge (DHS)

Gas retention time (GRT)

Hydraulic retention time (HRT)

Methane-oxidizing bacteria (MOB)
