# Supplementary material for: Environmental Factors Affecting the Community of Methane-oxidizing Bacteria
Source: Microbes Environ. 2022 Mar 26;37(1):ME21074. doi: 10.1264/jsme2.ME21074 (PMC8958294; doi:10.1264/jsme2.ME21074)
Supplement: Supplementary file 1 — Supplementary Material [file 37_21074_s1.pdf]

Supplemental material

Environmental factors affecting the community of methane-oxidizing bacteria

Hiromi Kambara[1], Takahiro Shinno[1], Norihisa Matsuura[2], Shuji Matsushita[3], Yoshiteru Aoi[4], Tomonori Kindaichi[5], Noriatsu Ozaki[5], Akiyoshi Ohashi[5]*.

Table S1 The actual environmental condition in the DHS reactors.

Run No.	Mean CH_4 concentration (%)		b/a×100 (%)	Mean pH	
	a: provided gas	b: off-gas		influent	effluent
1	9.80	8.46	86.33	7.31	6.81
2	0.10	0.08	83.43	7.30	6.60
3	9.56	8.59	89.84	7.31	6.94
4	9.79	7.78	79.44	3.89	3.55
5	9.90	8.45	85.32	7.46	7.11
6	0.10	0.09	86.56	7.48	7.20
7	9.62	8.42	87.55	7.46	7.22
8	9.95	7.83	78.77	4.27	3.87
9	0.01	0.01	93.51	7.32	6.97
10	0.01	0.01	96.25	7.32	7.07
11	74.61	73.27	98.20	4.15	3.87
12	9.20	7.98	86.68	4.14	4.00
13	0.01	0.01	96.20	7.33	7.08
14	0.10	0.09	82.69	7.33	7.05
15	9.60	8.50	88.49	5.44	4.81
16	9.30	7.69	82.63	6.37	5.78
17	0.11	0.08	73.14	4.21	4.05
18	1.20	0.80	66.72	5.12	4.86
19	4.77	3.64	76.23	5.10	4.94
20	72.25	69.90	96.75	7.35	7.04
21	0.10	0.09	84.37	4.14	3.95
22	1.04	0.70	67.24	5.11	4.79
23	1.05	0.81	77.11	6.11	6.07
24	74.60	73.56	98.60	7.32	7.02
25	9.33	7.58	81.22	5.20	4.80
26	9.33	7.62	81.60	6.19	5.85
27	0.10	0.09	84.09	5.10	5.01
28	0.10	0.08	80.63	6.19	5.85
29	9.50	8.79	92.48	4.01	3.97
30	9.50	8.37	88.10	7.35	6.99
31	0.11	0.09	88.00	4.04	3.98
32	0.11	0.09	88.91	7.34	7.07
33	1.15	0.89	77.59	5.22	5.05
34	0.10	0.09	85.49	4.02	3.96
35	0.10	0.10	95.06	7.13	6.92
36	0.10	0.10	96.37	6.86	6.75
37	9.63	8.05	83.58	7.21	7.11
38	9.63	8.77	91.09	6.81	6.70

Table S2 Relative abundance of read numbers in microbial analysis using primer sets of 341'f/805r, M γ 84f/341'r, and 341'f/M γ 705r for Run1 and Run5.

(a) Run1

Microorganisms (genus level)	Relative abundance (%)		
	341'f/805r	Mγ84f/341'r	341'f/Mγ705r
MOB	0.4	20.8	6.6
Methylomonas	0.2	20.8	6.6
Methylosarcina	0	0	0
Methylomagnum	0	0	0
Methyloparacoccus	0	0	0
Crenothrix	0.2	0	0
Others	99.6	79.2	93.4
Legionella	7.3	0	83.5
Legionella	0.7	21	0
Candidatus Berkiella	0.9	2.8	0.1
Candidatus Ovatusbacter	0.6	37.8	0
other	90.1	17.5	9.7
total	100	100	100

(b) Run5

Microorganisms (genus level)	Relative abundance (%)		
	341'f/805r	Mγ84f/341'r	341'f/Mγ705r
MOB	1.0	15.9	51.1
Methylomonas	0.3	1.9	16.8
Methylosarcina	0.3	13.8	10.7
Methylomonas	0.4	0	23.4
Methyloparacoccus	0	0	0.2
Crenothrix	0	0.2	0
Others	99	84.1	48.9
Legionella	0.5	0	24.2
Acidovorax	0.7	44.7	0
Candidatus Berkiella	0.9	35.5	3.1
Candidatus Ovatusbacter	0	0	0
other	96.6	3.9	21.7
total	100	100	100

Table S3 The used sequencers and the number of reads for each Run.

Run No.	16S rRNA gene		*pmoA* gene (Miseq)
	sequencer	reads	reads
1	Miseq	36613	16295
2	Roche/454	2527	9308
3	Miseq	34308	18563
4	Roche/454	2315	3513
5	Miseq	44135	17458
6	Roche/454	3152	10740
7	Miseq	36826	12991
8	Roche/454	1271	1580
9	Roche/454	2195	5052
10	Roche/454	3047	19179
11	Roche/454	3021	8186
12	Roche/454	2581	10910
13	Roche/454	2672	9598
14	Roche/454	1909	5053
15	Roche/454	2786	322
16	Miseq	31845	22632
17	Roche/454	2426	2777
18	Roche/454	2239	4609
19	Roche/454	2081	4163
20	Roche/454	3716	10095
21	Roche/454	3089	3821
22	Roche/454	2023	2171
23	Roche/454	3680	4830
24	Roche/454	3814	6354
25	Roche/454	3849	4383
26	Roche/454	3348	2788
27	Roche/454	1556	3271
28	Miseq	33169	8968
29	Roche/454	2542	4360
30	Roche/454	3385	11219
31	Roche/454	1575	8980
32	Roche/454	1809	9278
33	Miseq	34286	2599
34	Miseq	24044	5059
35	Miseq	31719	10391
36	Miseq	41405	13520
37	Miseq	39513	8256
38	Miseq	33032	9934

Table S4 Previous reports on MOB inhabited in various environmental conditions.

a) The effect of methane concentration on dominant MOB

pH	CH_4	Temperature (℃)	Nitrogen source	Active or dominant MOB	Primer set	probe	Reference
8.1	100%	20	150 mg NH_4Cl L^{-1}	Type I	A189f/mb661r	—	Hatamoto *et al*., 2010
8	200ppm	30	50 mg KNO_3 L^{-1}, 5mg NH_4Cl L^{-1}	Type II	A189f/A682r, A189f/mb661r	—	Hatamoto *et al*., 2011
5.5	200ppm	30	50 mg KNO_3 L^{-1}, 5mg NH_4Cl L^{-1}	Type II	A189f/A682r, A189f/mb661r	—	Hatamoto *et al*., 2011
5.64	11.2 mg L^{-1}	ND	0.5 mg NH_4Cl L^{-1}	Type I	341f/805r	—	Noorain *et al*., 2019
ND	High CH_4	ND	10 mM NH_4Cl or KNO_3	Type II	—	10γ, 9α	Amaral *et al*., 1995
ND	Low CH_4	ND	10 mM NH_4Cl or KNO_3	Type I	—	10γ, 9α	Amaral *et al*., 1995
ND	High CH_4	ND	1 mM $NaNO_3$	Type II (pure culture)	—	—	Graham *et al*., 1993
ND	Low CH_4	ND	1mM $NaNO_3$	Type I (pure culture)	—	—	Graham *et al*., 1993

ND: not described

b) The effect of temperature on dominant MOB

pH	CH_4	Temperature (℃)	Nitrogen source	Effect of temperature or dominant MOB	Primer set	probe	Reference
6.6	17.6 mg L^{-1}	26.7	27.2 mg N L^{-1} (NH_4^+)	Type I*	A189f/mb661	—	Matsuura *et al*., 2017
6.8	19.8 mg L^{-1}	12.2	28.0 mg N L^{-1} (NH_4^+)	Type II *	A189f/mb661	—	Matsuura *et al*., 2017
5.8–7.6	5%	3–20	ND	Type I increased at 5–10°C. Type II increased at 20°C.	—	—	Börjesson *et al*.,2004**
ND	85–100%	4–20	NO_3^-	Little	A189f/661Rev	MG705, MA445	Urmann *et al*., 2009
ND	4%	5–45	ND	High	A189f/A682r	—	Mohanty *et al*., 2007

* First closed DHS reactor

** MOB type was identified by PLFA analysis.

ND: not described

c) The effect of NH_4^+ on activity of MOB

pH	CH_4	Temperature (℃)	MOB activity	Primer set	Reference
6.8	0.3–4.2%	25	Type I MOB was enriched at higher NH_4^+ concentrations.	338f/805r	López *et al*., 2019
ND	10,000ppm	25	Type I was stimulated.<br>Type II was inhibited.	A189f/A682r	Mohanty *et al*., 2006
6.9–7.1*	4.9*	30	Type II dominance with only N_2 as a nitrogen source.	A189f/mb661r	Pfluger *et al*., 2011
6.8	500, 5,000 ppm	30	Methane oxidation rate of pure cultures decreased.	—	Nyerges *et al*., 2009
7	100 ppm	30	The extent of inhibition was similar to both types (pure culture).	—	King *et al*., 1994

* The conditions during Phase4.

ND: not described

d) MOB in nature environments

pH	location	Predominant MOB	Primer set	probe	Reference
4.2	Peat	Type II	—	Mcell-1026, Mcell-181, Mg84, Mg705, Ma221, Ma450	Dedysh *et al*., 2001
3.6–4.5	Peat	Type II	—	Msins-647, Msint-1268, Mcyst-1432, Mcaps-1032, MA-221, AcidM-181, M-450, Mcyst-1261, M-84, M-705, Mcell-1026	Dedysh *et al*., 2003
4.3	Forest	Type II	A189f/Forest675r, A189f/Gam634r	—	Kolb *et al*., 2005
7.7	Forest	Type I	A189f/Forest675r, A189f/Gam634r	—	Kolb *et al*., 2005
7.4–8.7	Oilsands tailings pond	Type I	926r/1392r, 189f/682r	—	Saidi-Mehrabad *et al*., 2013
4.4	Bog	Type II	P1/P2, Ar109f/Ar912r, 189f/682r, 945f/1401r	—	Gupta *et al*., 2012
7.1	Fen	Type I	P1/P2, Ar109f/Ar912r, 189f/682r, 945f/1401r	—	Gupta *et al*., 2012

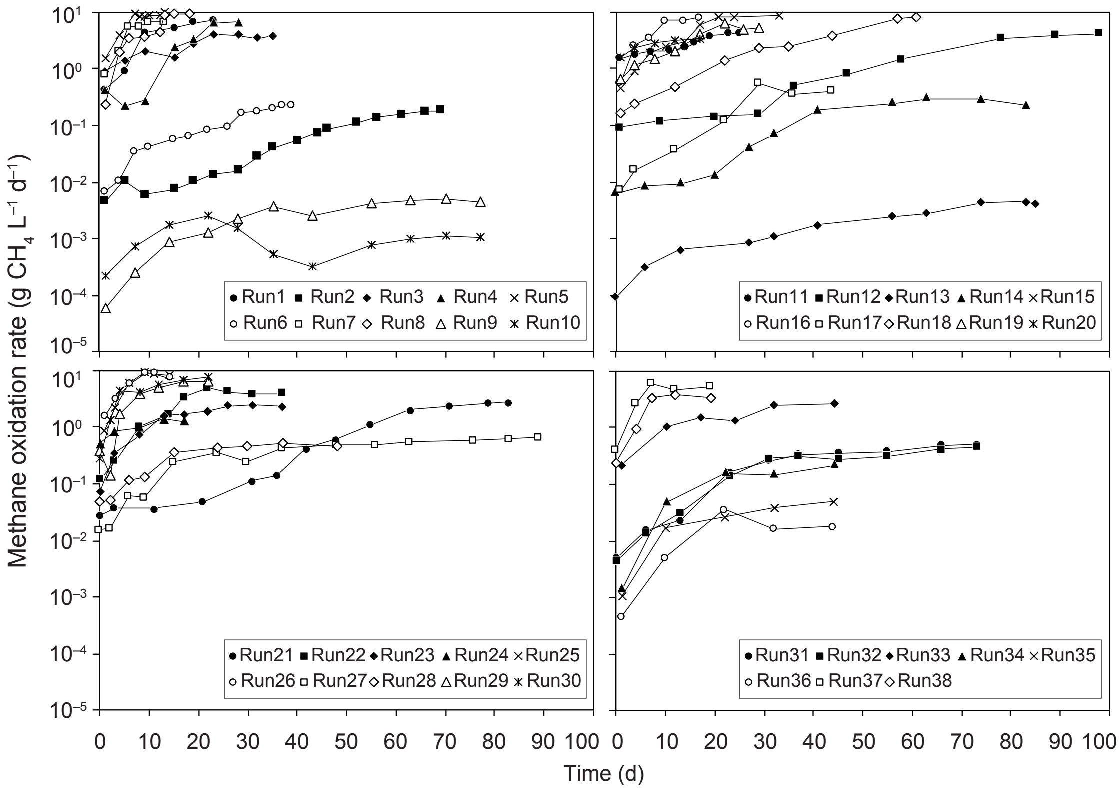

Fig. S1 Time courses in methane oxidation rate for all runs.

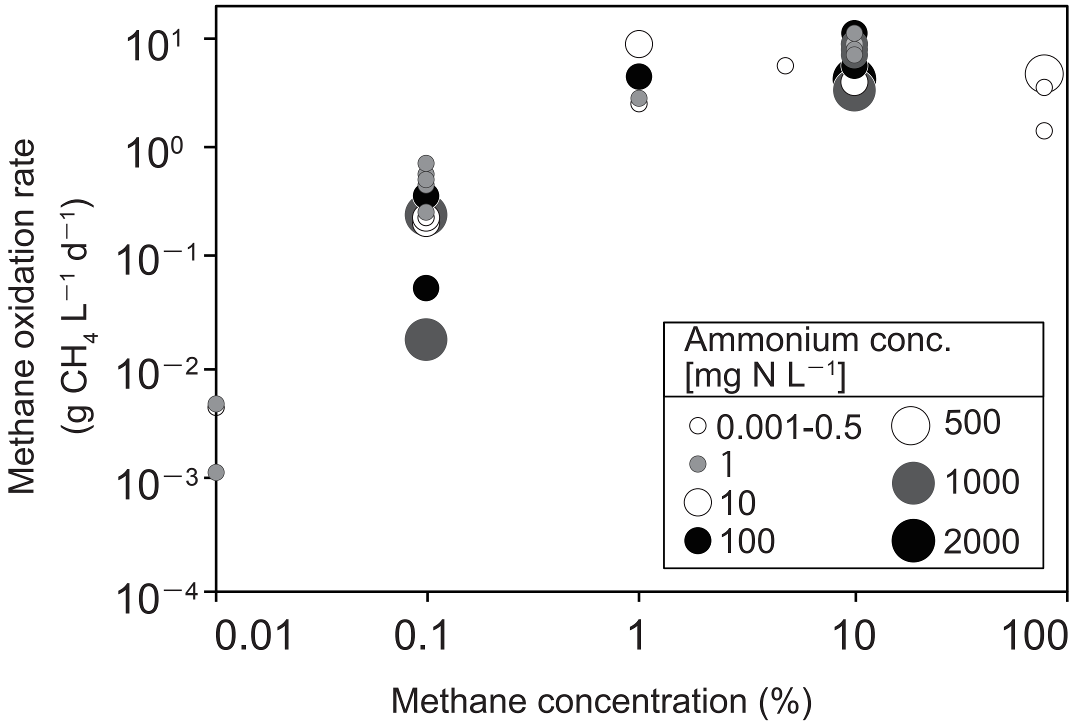

Fig. S2 Relationship between methane oxidation rate and supplied methane concentration.

Run 1	Run 2	Run 3	Run 4
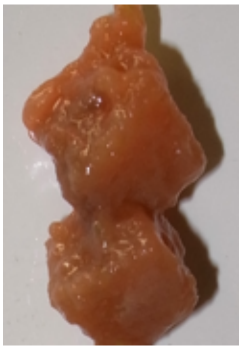	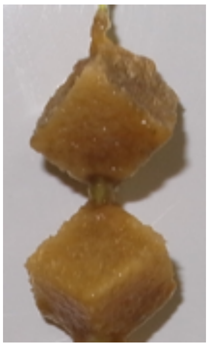	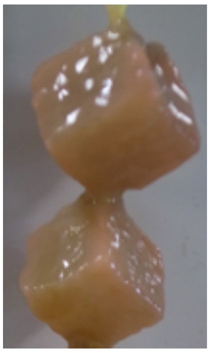	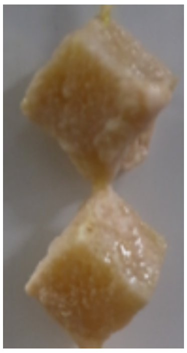
Run 5	Run 6	Run 7	Run 8
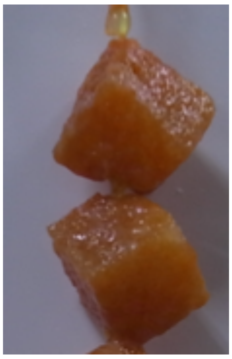	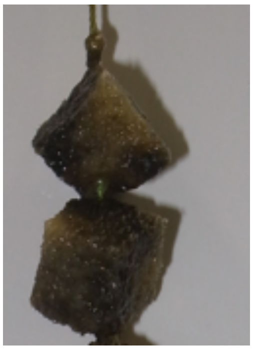	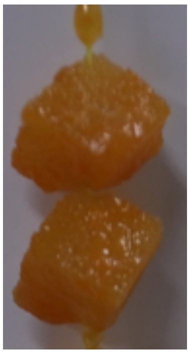	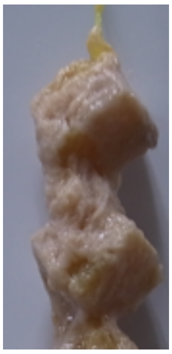
Run 9	Run 10	Run 11	Run 12
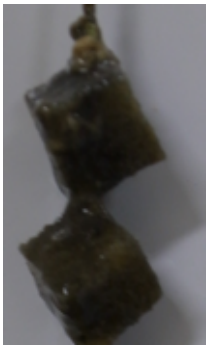	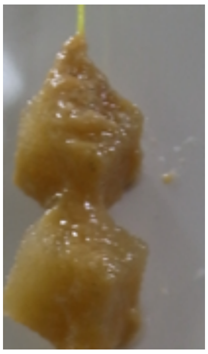	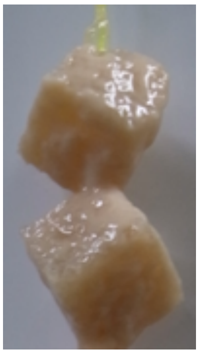	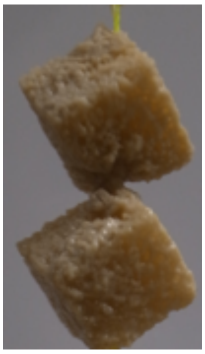
Run 13	Run 14	Run 15	Run 16
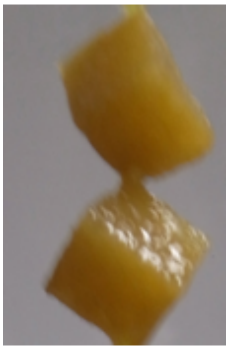	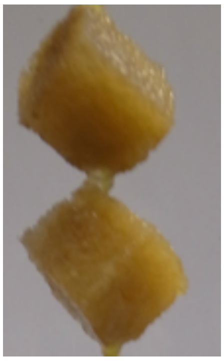	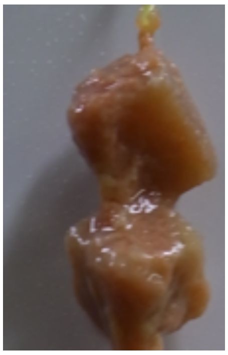	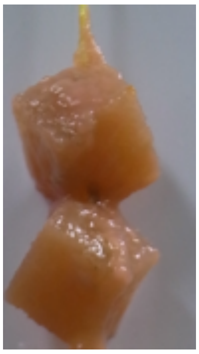

(continued)

Run 17	Run 18	Run 19	Run 20
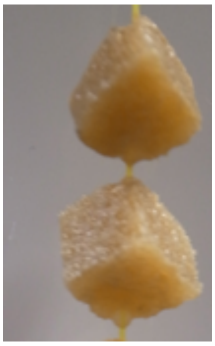	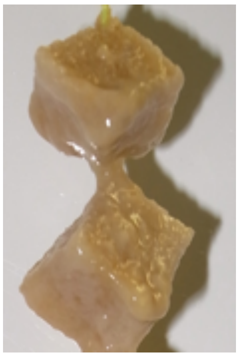	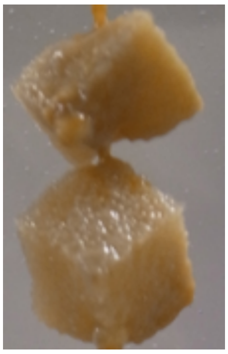	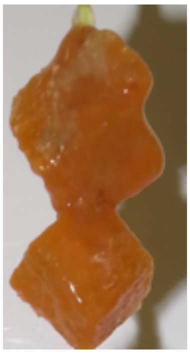
Run 21	Run 22	Run 23	Run 24
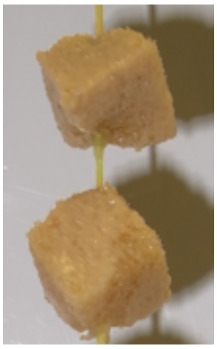	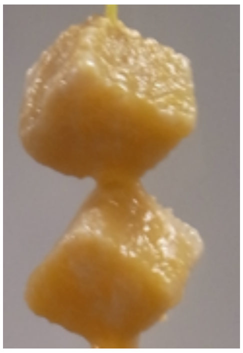	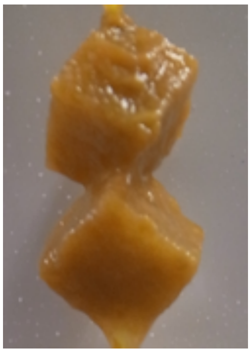	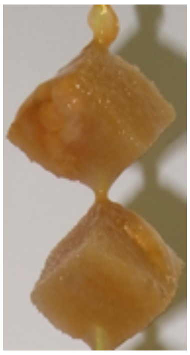
Run 25	Run 26	Run 27	Run 28
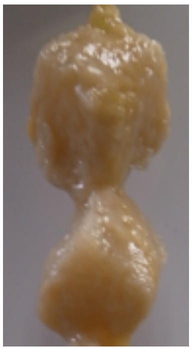	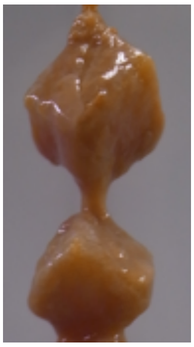	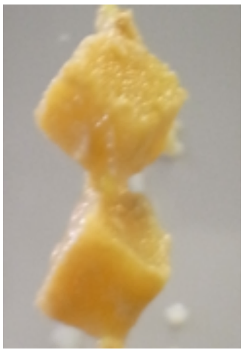	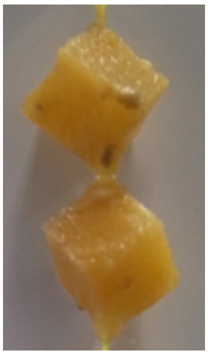
Run 29	Run 30	Run 31	Run 32
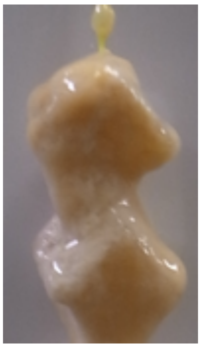	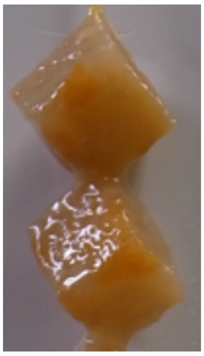	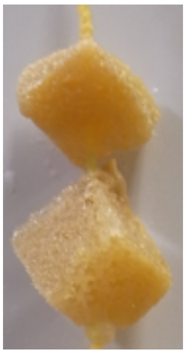	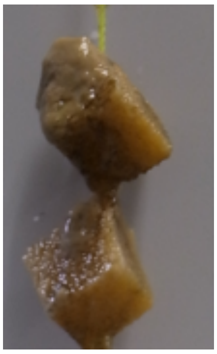

(continued)

Run 33	Run 34	Run 35	Run 36
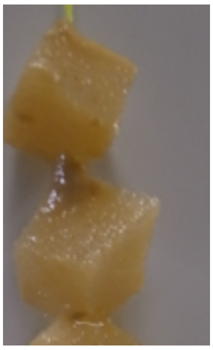	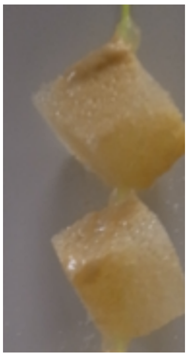	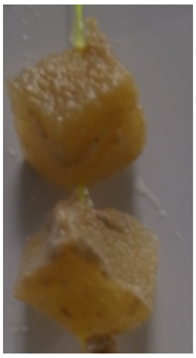	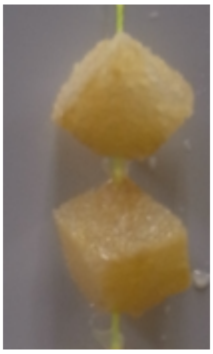
Run 37	Run 38		
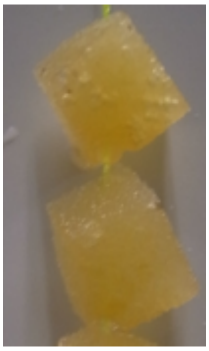	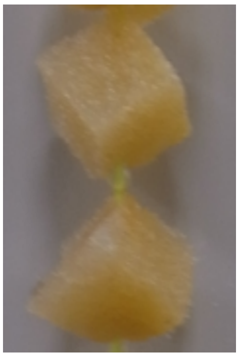		

Fig. S3 Biomass observed in each reactor at the end of cultivated period.

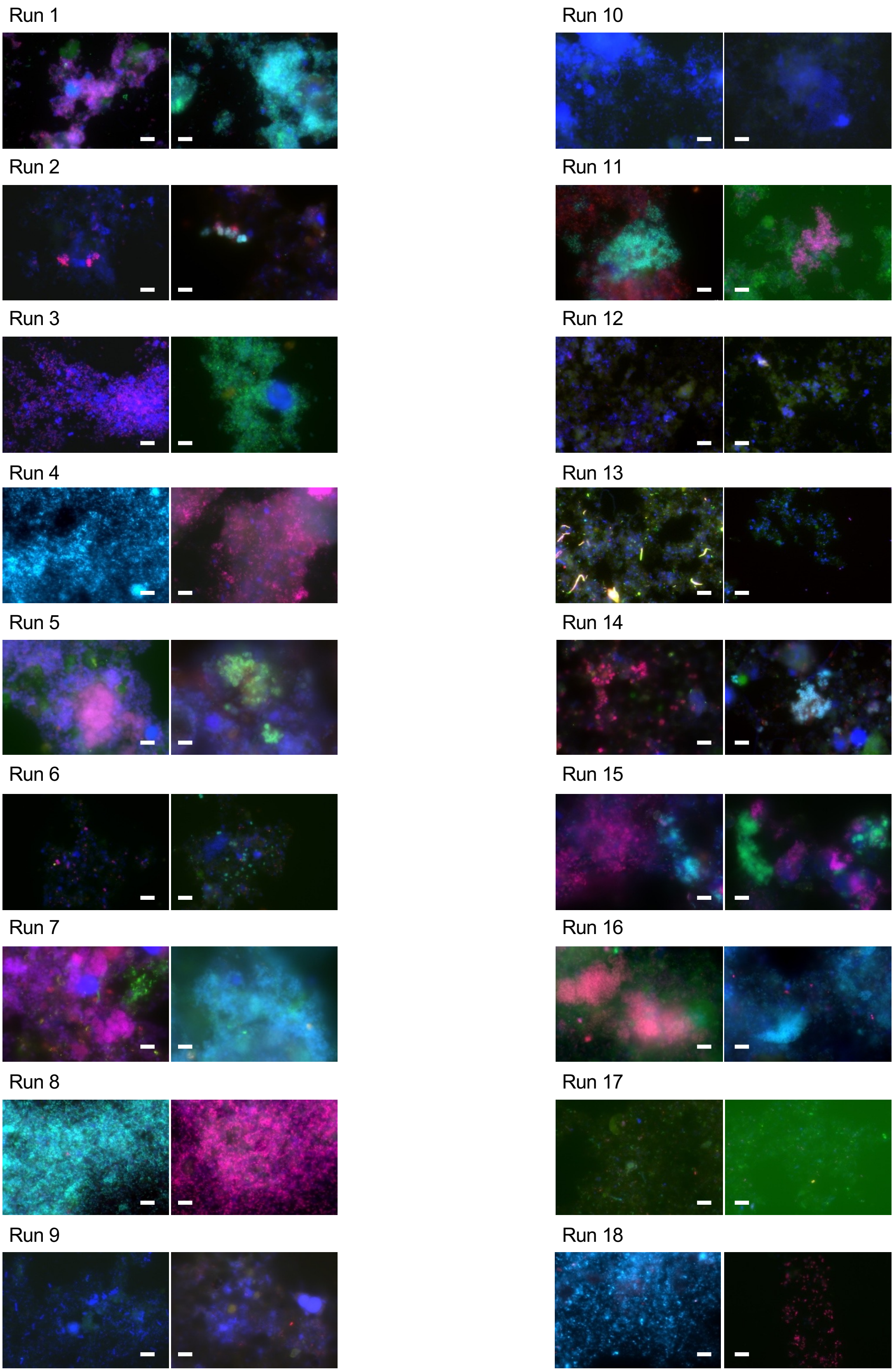
Run 1
Run 2
Run 3
Run 4
Run 5
Run 6
Run 7
Run 8
Run 9
Run 10
Run 11
Run 12
Run 13
Run 14
Run 15
Run 16
Run 17
Run 18

(continued)

Run 19

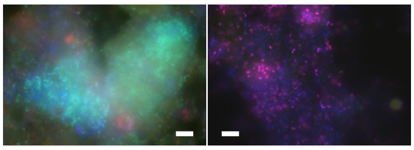

Run 20

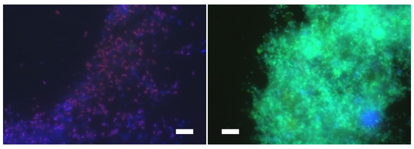

Run 21

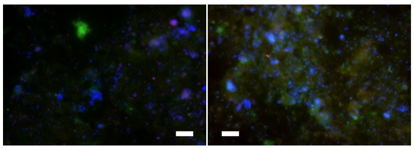

Run 22

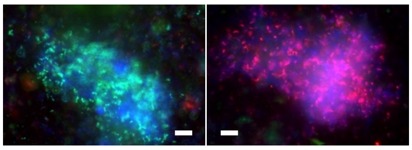

Run 23

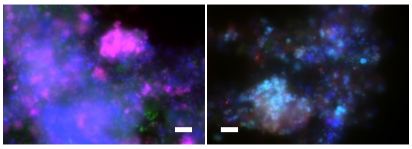

Run 24

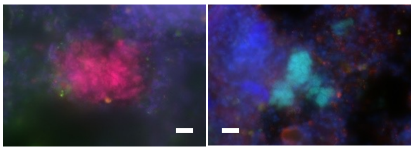

Run 25

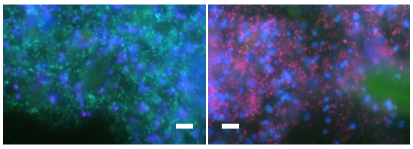

Run 26

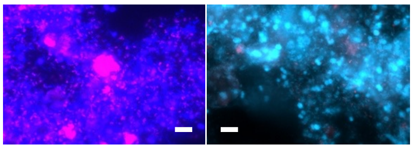

Run 27

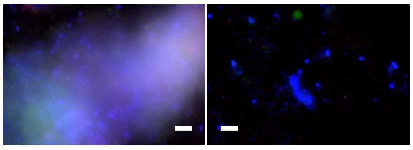

Run 28

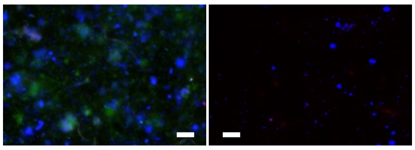

Run 29

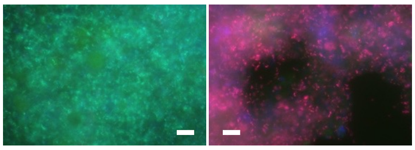

Run 30

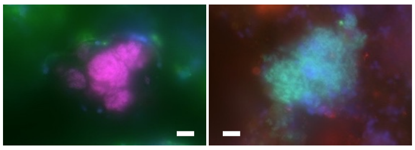

Run 31

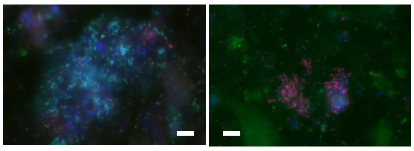

Run 32

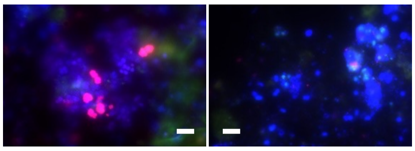

Run 33

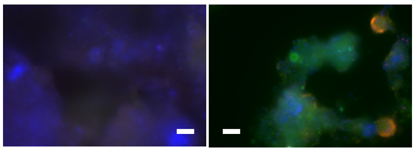

Run 34

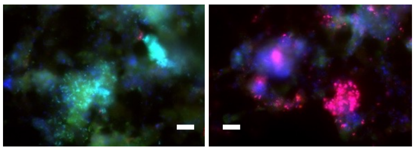

Run 35

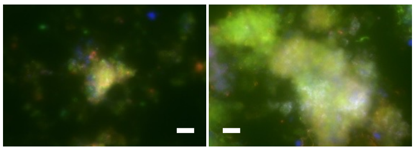

Run 36

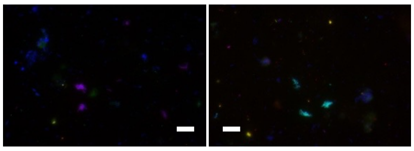

(continued)

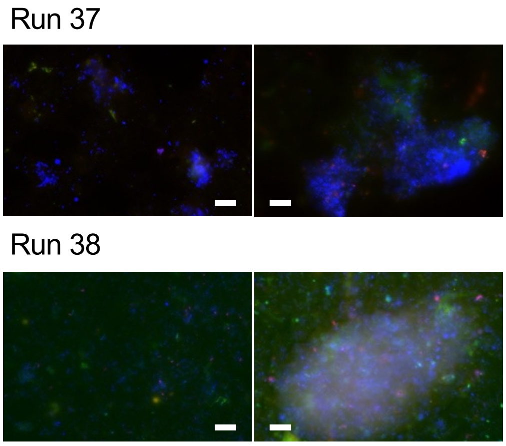

Fig. S4 FISH images combined from three images, which were gained from the fluorescence of Alexa 647, Alexa 555, and Alexa 488. The combination of probe with fluorescence is Alexa 647 with EUB mix (blue), Alexa 555 with Mγ84 and Mγ705 probes (red) for type I MOB, Alexa 488 with Mα450 probes (green) for type II MOB (left). The combination was changed (right). Scale bars represent 10 μm.

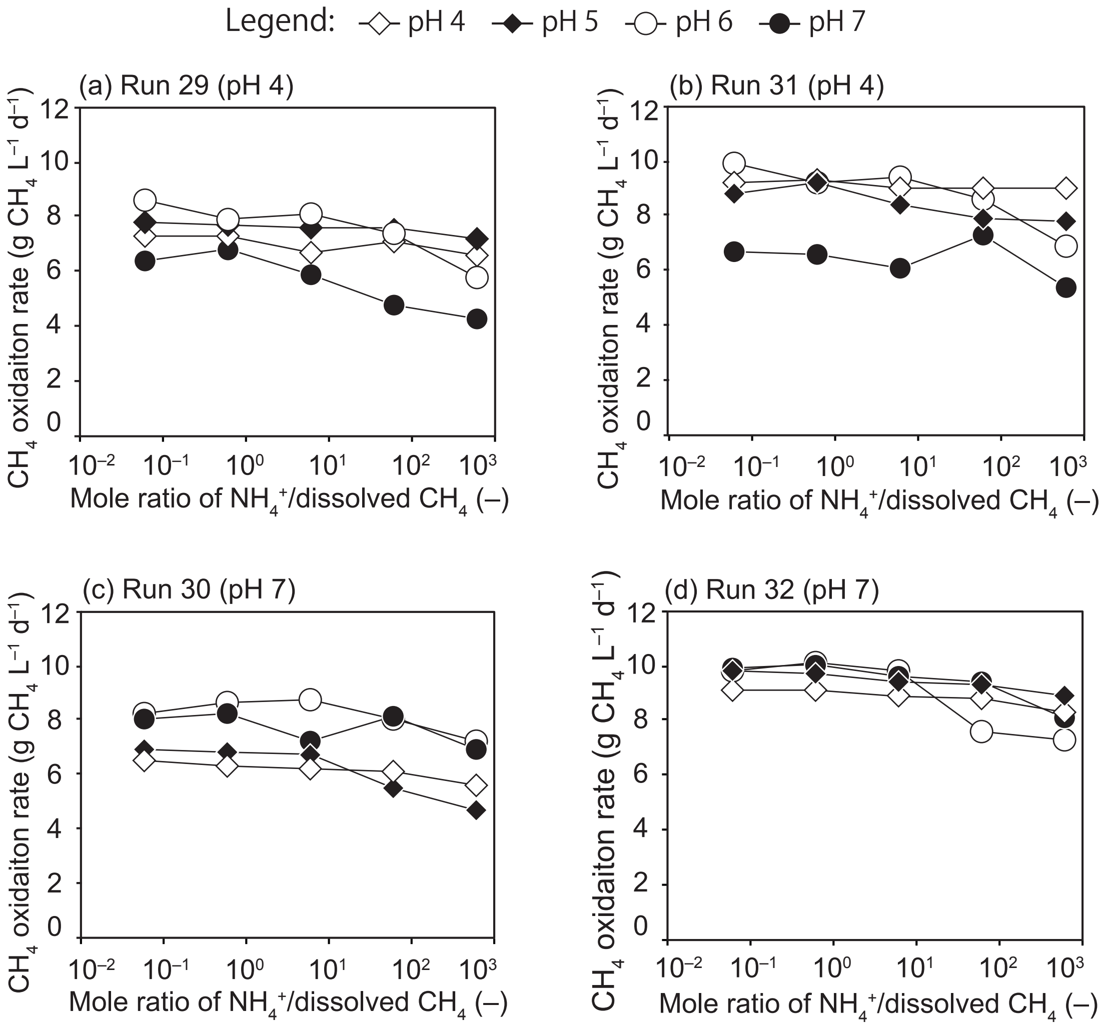

Fig. S5 Effect of the mole ratio of NH_4^+/dissolved CH_4 on methane oxidation rate at 10% CH_4 under different pH conditions for the samples of Runs 29 to 32. The pH in the parentheses indicates the original pH value during the enrichment.

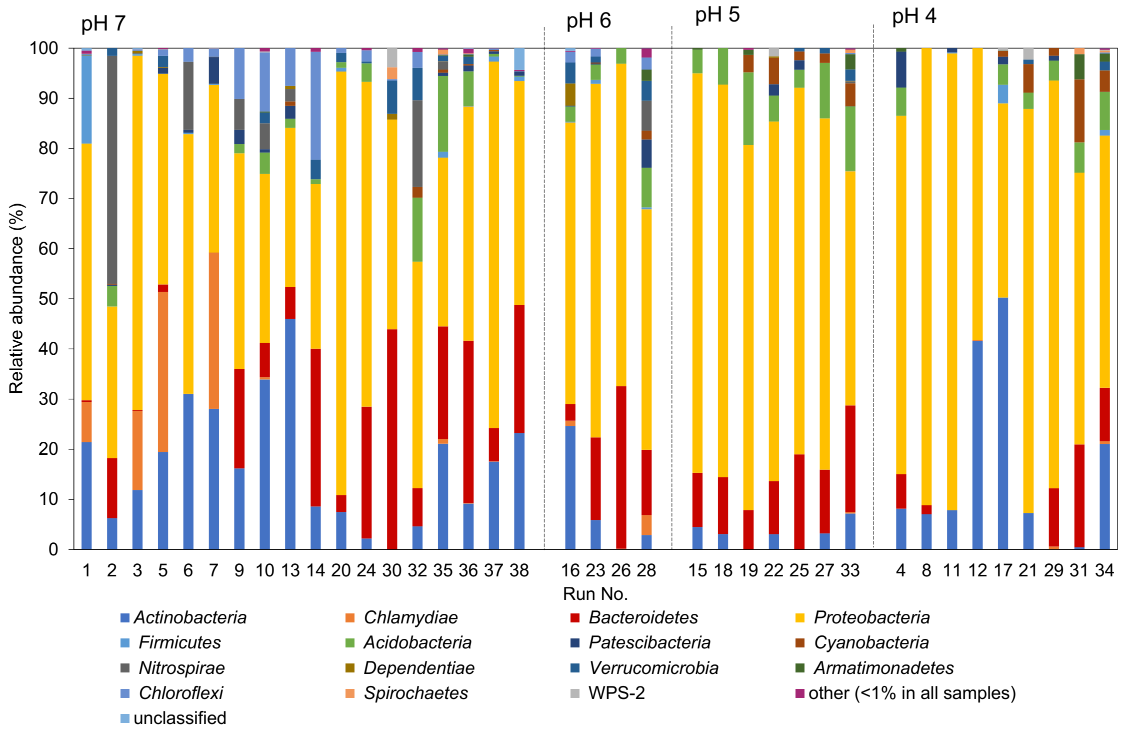

Fig. S6 Relative abundance based on 16S rRNA genes.

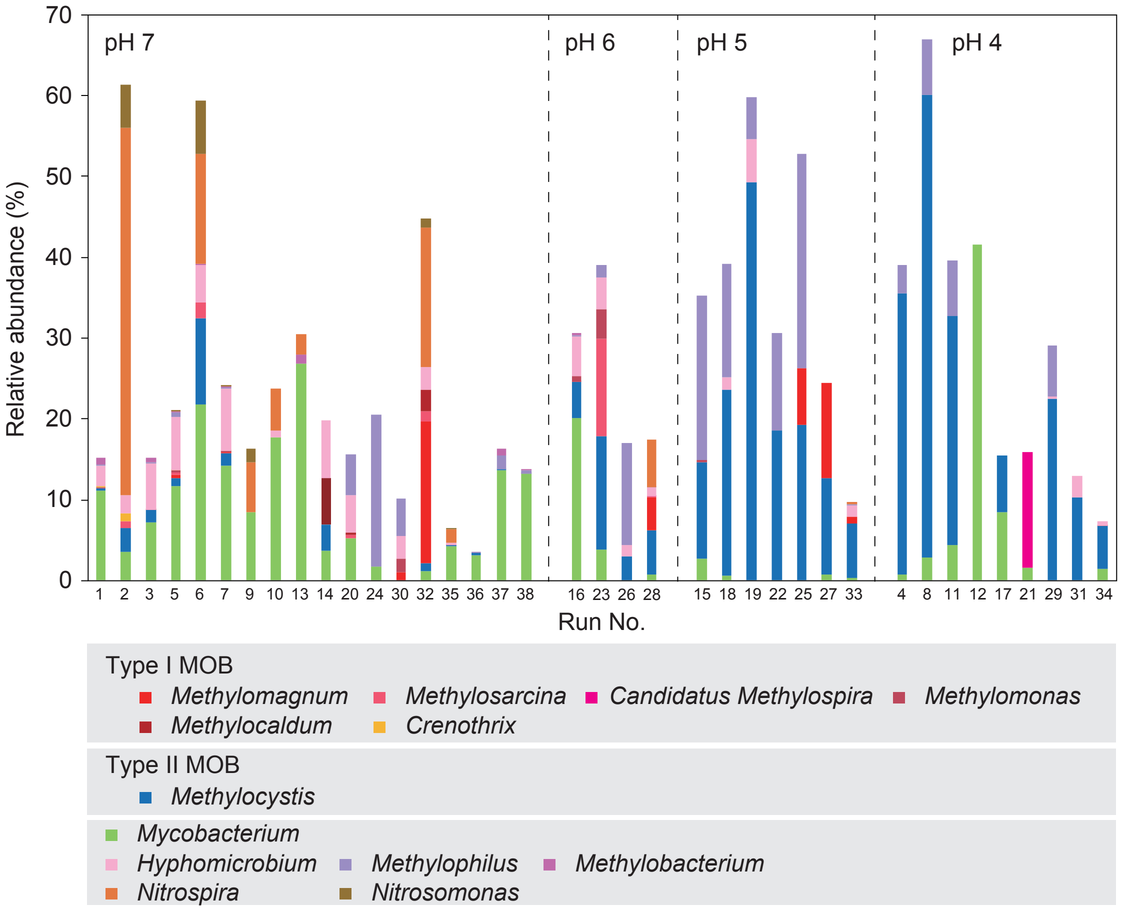

Fig. S7 Relative abundance of MOB, Methylotrophs, *Mycobacterium*, and Nitrifiers based on 16S rRNA genes.

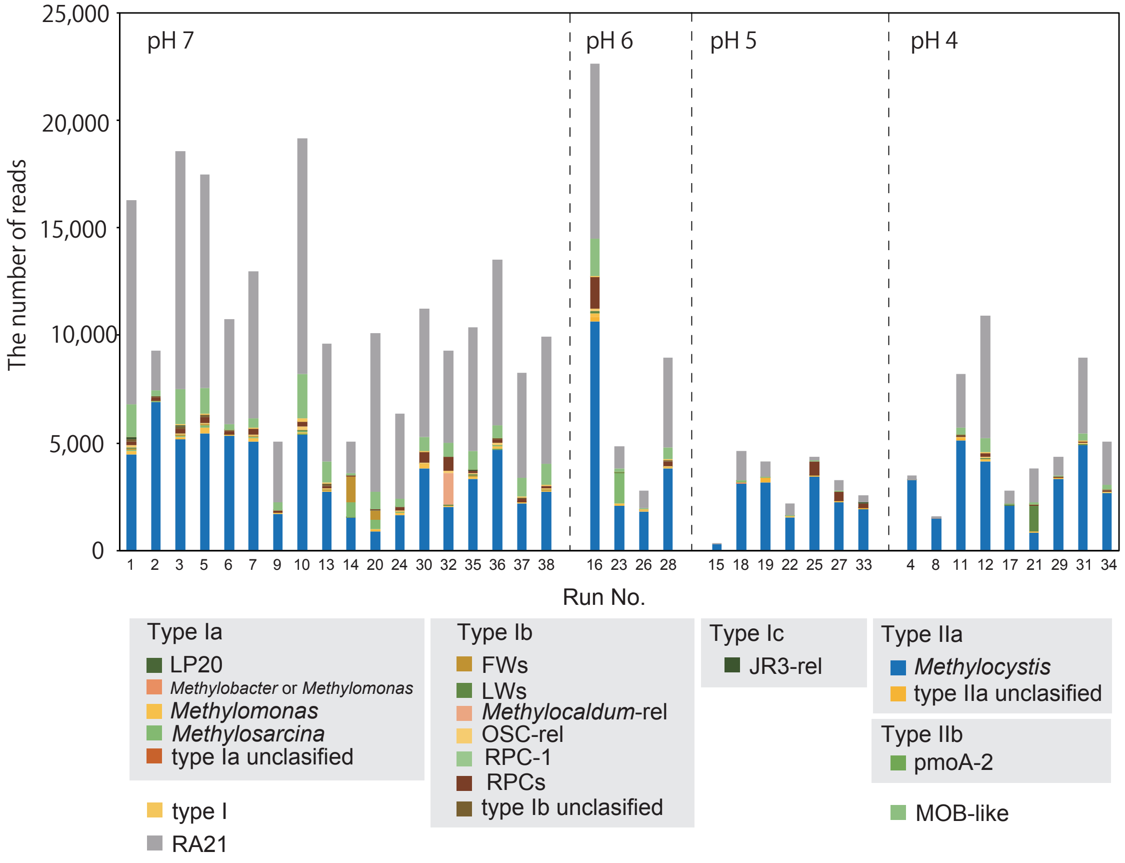

Fig. S8 The number of reads based on *pmoA* genes.

References

Amaral, J.A., Archambault, C., Richards, S.R., Knowles, R. (1995) Denitrification associated with group I and II methanotrophs in a gradient enrichment system. *FEMS Microbiol Ecol* **18**: 289–298.

Börjesson, G., Sundh I., Svensson, B. (2004) Microbial oxidation of CH_4 at different temperatures in landfill cover soils. *FEMS Microbiol Ecol* **48**: 305–312.

Dedysh, S.N., Derakshani, M., Liesack, W. (2001) Detection and enumeration of methantrophs in acidic sphagnum peat by 16S rRNA fluorescence in situ hybridization, including the use of newly developed oligonucleotide probes for Methylocella palustris. *Appl Environ Microbiol* **67**: 4850–4857.

Dedysh, S.N., Dunfield, P.F., Derakshani, M., Stubner, S., Heyer, J., Liesack, W. (2003) Differential detection of type II methanotrophic bacteria in acidic peatlands using newly developed 16S rRNA-targeted fluorescent oligonucleotide probes. *FEMS Microbiol Ecol* **43**: 299–308

Graham, D.W., Chaudhary, J.A., Hanson, R.S., Arnold, R.G. (1993) Factors affecting competition between type I and type II methanotrophs in two-organism, continuous-flow reactors. *Microb Ecol* **25**: 1–17.

Gupta, V., Smemo, K.A., Yavitt, J.B., Basiliko N. (2012) Active methanotrophs in two contrasting north American peatland ecosystems revealed using DNA-SIP. *Microb Ecol* **63**: 438–445.

Hatamoto, M., Yamamoto, H., Kindaichi, T., Ozaki, N., Ohashi, A. (2010) Biological oxidation of dissolved methane in effluents from anaerobic reactors using a down-flow hanging sponge reactor. *Water Res* **44**: 1409–1418.

Hatamoto, M., Koshiyama, Y., Kindaichi, T., Ozaki, N., Akiyoshi, O. (2011) Enrichment and identification of methane-oxidizing bacteria by using down-flow hanging sponge bioreactors under low methane concentration. *Ann Microbiol (London, U. K.)* **61**: 683–687.

King, G.M., and Schnell, S. (1994) Ammonium and nitrite inhibition of methane oxidation Methylobacter albus BG8 and Methylosinus trichosporium OB3b at low methane concentrations. *Appl Environ Microbiol* **60**: 3508–3513.

Kolb, S., Knief, C., Dunfield, P.F., Conrad, R. (2005) Abundance and activity of uncultured methanotrophic bacteria involved in the consumption of atmospheric methane in two forest soils. *Environ Microbiol* **7**: 1150–1161.

López, J.C., Porca, E., Collins, G., Clifford, E., Quijano, G., Muñoz, R. (2019) Ammonium influences kinetics and structure of methanotrophic consortia. *Waste Manage. (Oxford, U. K.)* **80**: 345–353.

Matsuura, N., Hatamoto, M., Yamaguchi, T., Ohashi, A. (2017) Methanotrophic community composition based on pmoA genes in dissolved methane recovery and biological oxidation closed downflow hanging sponge reactors. *Biochem Eng J* **124**: 138–144.

Mohanty, S.R., Bodelier, P.L.E, Floris, V., Conrad, R. (2006) Differential effects of nitrogenous fertilizers on methane-consuming microbes in rice field and forest soils. *Appl Environ Microbiol* **72**: 1346–1354.

Mohanty, S.R., Bodelier, P.L.E., Conrad, R. (2007) Effect of temperature on composition of the methanotrophic community in rice field and forest soil. *FEMS Microbiol Ecol* **62**: 24–31.

Noorain, R., Kindaichi, T., Ozaki, N., Aoi, Y., Ohashi, A. (2019) Integrated biological-physical process for biogas purification effluent treatment. *J Environ Sci (Beijing, China)* **83**: 110–122.

Nyerges, G., and Stein, Y.L. (2009) Ammonia cometabolism product inhibition vary considerably among species of methanotrophic bacteria. *FEMS Microbiol Lett* **297**: 131–136.

Pfluger, A.R., Wu, W., Pieja, A.J., Wan, J., Rostkowski, K.H., Criddle, C.S. (2011) Selection of Type I and Type II methanotrophic proteobacteria in a fluidized bed reactor under non-sterile conditions. *Bioresour Technol* **102**: 9919–9926.

Saidi-Mehrabad, A., He, Z., Tamas, I., Sharp, C.E., Brady, A.L., Rochman, F.F., Bodrossy, L., Abell, G.C. *et al*. (2013) Methanotrophic bacteria in oilsands tailings ponds of northern Alberta. *ISME J* **7**: 908–921.

Urmann, K., Lazzaro, A., Gandolfi, I., Schroth, M.H., Zeyer, J. (2009) Response of methanotrophic activity and community structure to temperature changes in a diffusive CH_4/O_2 counter gradient in an unsaturated porous medium. *FEMS Microbiol Ecol* **69**: 202–212.
